# Expression of luteinizing hormone receptor during development of bovine fetal ovary

**DOI:** 10.1590/1984-3143-AR2023-0112

**Published:** 2024-04-15

**Authors:** Alan Brunholi Giroto, Marina Platzeck Chaves, Priscila Helena dos Santos, Patrícia Kubo Fontes, Sarah Gomes Nunes, Thainá Sallum Bacco Manssur, Leonardo Oliveira Mendes, Anthony César de Souza Castilho

**Affiliations:** 1 Universidade do Oeste Paulista, Presidente Prudente, SP, Brasil; 2 Departamento de Farmacologia, Instituto de Biociências, Universidade Estadual Paulista, Botucatu, SP, Brasil

**Keywords:** LHCGR, preantral follicle, steroidogenesis, microRNAs

## Abstract

Steroids and gonadotrophins are essential for the regulation of late stages of preantral development and antral follicular development. Although the luteinizing hormone receptor (LHCGR) has been detected in the preantral follicles of rats, rabbits, and pigs, its expression, in bovine fetal ovary, has not been demonstrated. Based on this, we aimed to investigate the expression of the *LHCGR* and *LHCGR* mRNA binding protein (*LRBP*), as well as, to quantify bta-miR-222 (a regulatory microRNA of the LHCGR gene) during the development of bovine fetal ovary. In summary, *LHCGR* expression was observed in the preantral follicle in bovine fetal ovary, from oogonias to primordial, primary and secondary stages, and the mRNA abundance was lower on day 150 than day 60. However, the mRNA abundance of *LRBP* followed the opposite pattern. Similar to LRBP, the abundance of bta-miR-222 was higher on day 150 than day 60 or 90 of gestation. The LHCGR protein was detected in oogonia, primordial, primary, and secondary follicles. Moreover, both oocytes and granulosa cells showed positive immunostaining for LHCGR. In conclusion, we suggest the involvement of LHCGR/LRBP/bta-mir222 with mechanisms related to the development of preantral follicles in cattle.

## Introduction

In cattle, all preantral follicular stages occur in the fetal ovary during pregnancy (Erickson, 1966). Although fetal ovarian development occurs independently of gonadotrophins, these hormones do influence this process (Fortune and Eppig, 1979). As follicle stimulating hormone (FSH) receptors (FSHR) have been detected in bovine primary follicles (Wandji et al., 1996), the primary role of FSH at this stage of development cannot be excluded (Gutiérrez et al., 1997; Webb et al., 2003). There is a study that reports the expression of *LHCGR* in different preantral follicles during fetal ovarian development in bovine species; *LHCGR* was highly expressed early in gestation, decreased in mid-gestation and then increased levels in the final trimester of pregnancy (Hatzirodos et al., 2019).

Luteinizing hormone (LH) plays a key role in the control of physiological processes in the ovary, such as the development of antral follicles and ovulation (Xu et al., 1995). In antral follicles, the LH receptors have been detected in theca cells of healthy follicles and, subsequently, in granulosa cells (Ereno et al., 2015; Xu et al., 1995). The possible mechanisms involved in the LHCGR regulation of granulosa cells include the LH receptor binding protein (LRBP) and certain microRNAs. LRBP is a mRNA binding protein that binds to the LHCGR (*LHCGR* gene) coding region and represses its translation (Nair et al., 2002). In cattle, (Ereno et al., 2015) first demonstrated the inverse correlation between *LRBP* expression and *LHCGR* mRNA regulation at the time of follicular deviation in granulosa cells.

Similarly, recent studies demonstrated that the post-transcriptional regulation of *LHCGR* by miRNA occurs in the ovary of several species (Gilchrist et al., 2016; Menon et al., 2013); this includes miR-222, which was suggested by (Hossain et al., 2009) as a possible regulator of *LHCGR* expression. (Salilew-Wondim et al., 2014) investigated the expression of miR-222 in the theca and granulosa cells of bovine antral follicles and reported lower expression in the granulosa cells from dominant follicles. In addition, our group, (Santos et al., 2018) reported lower miR-222 expression in granulosa cells from superstimulated cows submitted to ovarian superstimulation than control animals and inverse proportionality to the abundance of *LHCGR* mRNA.

The steroid hormones and their actions are directly dependent on the expression of gonadotrophic receptors and steroidogenic enzymes in follicular somatic cells (Fortune et al., 2001). Although there is evidence that the developing gonads produce steroids throughout gestation (Shemesh et al., 1978; Tanaka et al., 2001), probably by using the same sequential enzymatic processing of the adult ovary (Conley and Bird, 1997), the role of steroids during the development of bovine ovaries has not been sufficiently clarified. Thus, in the present study, we aimed to localize and quantify the LHCGR and LRBP expression in the fetal ovary and to measure expression of bta-miR-222 in the developing bovine fetal ovary.

## Methods

### Tissues

In accordance with the methods of (Tanaka et al., 2001) and (Castilho et al., 2014), 20 female fetuses at 60, 90, 120, and 150 days of gestational (6–8, 13.6–15.6, 23.4–25.4, and 37–40; respectively), predominantly from Nellore cattle (*Bos Taurus indicus*), were obtained from a local abattoir near the University of São Paulo State campus at Assis city and were classified according to specific crown-rump lengths intervals (n = 5/group). Subsequently, one fetal ovary of each fetus was transported to the laboratory in TRIzol^®^ Reagent for RNA extraction and the other was transported in methacarn solution (60% methanol, 30% chloroform, 10% acetic acid) for histology and immunohistochemistry.

#### Gene expression

For total RNA extraction, the whole fetal ovaries were homogenized (Precellys^®^, Bertin Technologies) and later subjected to the TRIzol^®^ protocol (Invitrogen^®^, São Paulo, Brazil) to obtain total RNA in accordance with the manufacturer’s instructions. The total RNA concentration was measured by spectrophotometry using a Nanodrop (ND-2000^®^). The total RNA from samples (1μg) was incubated with DNAse (1 U/μg; Invitrogen, Carlsbad, CA, USA) and then reverse transcribed by using a random primer in accordance with protocol provided by High Capacity Kit instructions (Applied Biosystems, Foster City, CA, USA).

The RT-qPCR analysis for each gene (*LHCGR* and *LRBP*) were performed by QuantStudio™ 7 Flex using Power Sybr^®^ Green PCR Master Mix system (Applied Biosystems). The mRNA abundance of target genes was assessed in a total reaction volume of 25 μL, with 1.0 μL of each sample and 24 μL of probe plus primers in accordance with the methods of (Machado et al., 2009) for endogenous genes, and (Castilho et al., 2015; Ereno et al., 2015) for the target genes. The thermal cycling conditions comprised 95°C for 10 min, followed by 40 cycles of denaturing at 95°C for 10 s, annealing, and extension for 1 min, with different temperatures used for different genes. There actions were optimized to provide the maximum amplification efficiency for each gene. The specificity of each PCR product was determined by melting curve analysis. Each sample was analyzed in duplicate and negative controls were run for each plate.

To choose the most stable reference gene for the detailed analyses of fetal ovaries, the amplification profiles of peptidylprolyl isomerase A (*PPIA*), glyceraldehyde-3-phosphate dehydrogenase (*GAPDH*), and histone H2AFZ (*H2AFZ*) were compared by using the geNorm applet for Microsoft Excel (medgen.ugent.be/genorm; (Ramakers et al., 2003)). The most stable housekeeping gene was *PPIA*. The ∆∆Ct method with efficiency correction was used to calculate relative expression values (target genes/*PPIA*) for each target gene; the mean value for the day 60 group was used as a calibrator (Pfaffl, 2001).

#### miRNA expression

The miRNA extraction was performed from 50 μg of total RNA by using a mirVana™ miRNA Isolation Kit (Life Technologies^®^, Carlsbad, USA) in accordance with the manufacturer’s instruction and subsequently stored at -80°C. To reverse transcribe target miRNAs (bta-miR-222, Has-miR-191, and RNU-43), we used TaqMan^®^ Reverse Transcription Reagents (Applied Biosystems, Foster City, CA, USA) in accordance with the manufacturer’s protocols. The qPCR analyses were performed by QuantStudio™ 7 Flex using TaqMan^®^ Universal PCR Master Mix. The final volume of PCR mix was 20 μL and the cycling conditions were 95°C for 10 min, for enzyme activation, followed for 40 cycles of denaturation (95ºC for 15 s) and annealing/extension (60°C for 60 s). All samples were run in duplicate.

To quantify the relative abundance of bta-miR-222, we used the geometric mean of the expression of RNU43 and has-miR-191 as a reference. The ∆∆Ct method with efficiency correction was used to calculate the relative expression value (bta-miR-222/RNU43_has-miR-191 geometric mean) with the mean value on day 60 used as the calibrator (Pfaffl, 2001).

#### Immunohistochemistry

We fixed the tissues from all groups in methacarn for 6h and stored in 70% ethanol. Tissue dehydration was performed by using a series of graded ethanol solutions and the dehydrated tissues were embedded in Paraplast^®^ (Oxford Labware, St. Louis, MO, USA). The blocks were sectioned into 4 μm thick slices and the sections were placed on poly-L-lysine–coated slides, which were deparaffinized and washed. One ovary at each estimated fetal age was subjected to immunohistochemical staining to localize LHR (Rabbit polyclonal - ab96603, Abcam, Cambridge, UK).

The antigens were retrieved at high temperature (100 °C) for 30 min in 10 mM citrate buffer (pH 6.0). Endogenous peroxidase activity was quenched through incubation with 3% H_2_O_2_ diluted in methanol for 15 min and nonspecific protein binding was blocked by the incubation of the slides in bovine serum albumin (BSA), diluted to 3% in PBS, plus 0.1% NP-40. The primary antibodies anti-hCG receptor (ab96603, Abcam, 1/100) was diluted in 1% BSA in PBS plus 0.1% NP-40 and the slides were incubated overnight in this solution at 4 °C. For the immunoperoxidase assay, the slides were rinsed in PBS, incubated with secondary antibody biotinylated Goat Anti-Rabbit IgG H&L (ab97049, Abcam), followed by a VECTASTIN ABC Kit (Vector Laboratories Ltd), and visualized with by using diaminobenzidine. The protocol used was standardized by Mendes et al. (2015). The sections were counterstained with Harris hematoxylin. The LHR immunostaining was performed in the corpus luteum of adult bovine ovary (positive control) to confirm the quality of the primary antibody. The negative control was performed in the absence of the primary antibody.

### Statistical analysis

Once all data were normally distributed, we used ANOVA to test for effects of gestational days on the relative abundance of miRNA and target genes. The differences between means were determined by the Tukey test. The analyses were computed by using JMP software (SAS Institute Cary, NC). The data are presented as the mean ± SEM; and differences were considered significant for P≤0.05.

## Results

**Figure 1:**
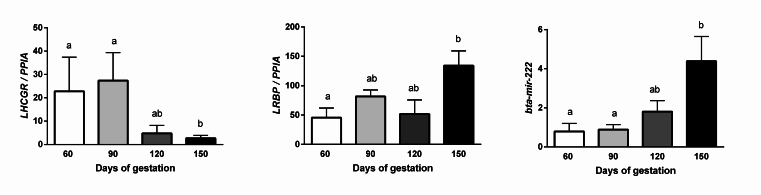
Transcriptional abundance of *LHCGR*, *LRBP*, and *bta-miR222* in the days 60, 90, 120 and 150 of gestation. To choose the most stable reference gene for the detailed analyses of fetal ovaries, the amplification profiles of peptidylprolyl isomerase A (*PPIA*), glyceraldehyde-3-phosphate dehydrogenase (*GAPDH*), and histone H2AFZ (*H2AFZ*) were compared by using the geNorm. The most stable housekeeping gene was PPIA. The ∆∆Ct method with efficiency correction was used to calculate relative expression values (target genes/PPIA) for each target gene; the mean value for the day 60 group was used as a calibrator. The data are presented as the mean (± S.E.M.). Bars with different letters are significantly different (P≤0.05).

In general, we observed the expression of the target genes and bta-miR-222 in bovine fetal ovary during gestation. When the effect of gestational day was investigated, the abundance of *LHCGR* mRNA was lower on day 150 of gestation than on day 60 and 90 (P=0.04); in contrast, the mRNA abundance of *LRBP* was higher on day 150 than on day 60 and 90 (P=0.03). Similar to *LRBP*, the expression of bta-miR-222 was also higher on day 150 than days 60 and 90 (P=0.02; Figure 1).

**Figure 2:**
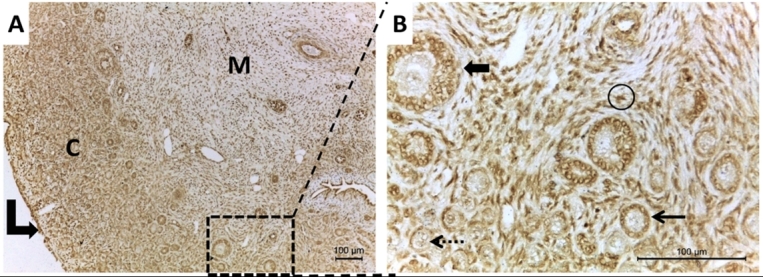
Representative photomicrographs showing immunolocalization of LHR protein in day 150 bovine fetal ovary (A and B). Overview LHR staining in cortex (C) and medulla (M) with immunostaining in the ovarian surface (curve arrow) (A). The ovarian cortex showed granulosa LHR positive cells in secondary (thick arrow), primary (thin arrow) and primordial (dashed arrow) follicles as well as stromal positive cells (circle) (B). Bars: A and B: 100 μm.

**Figure 3:**
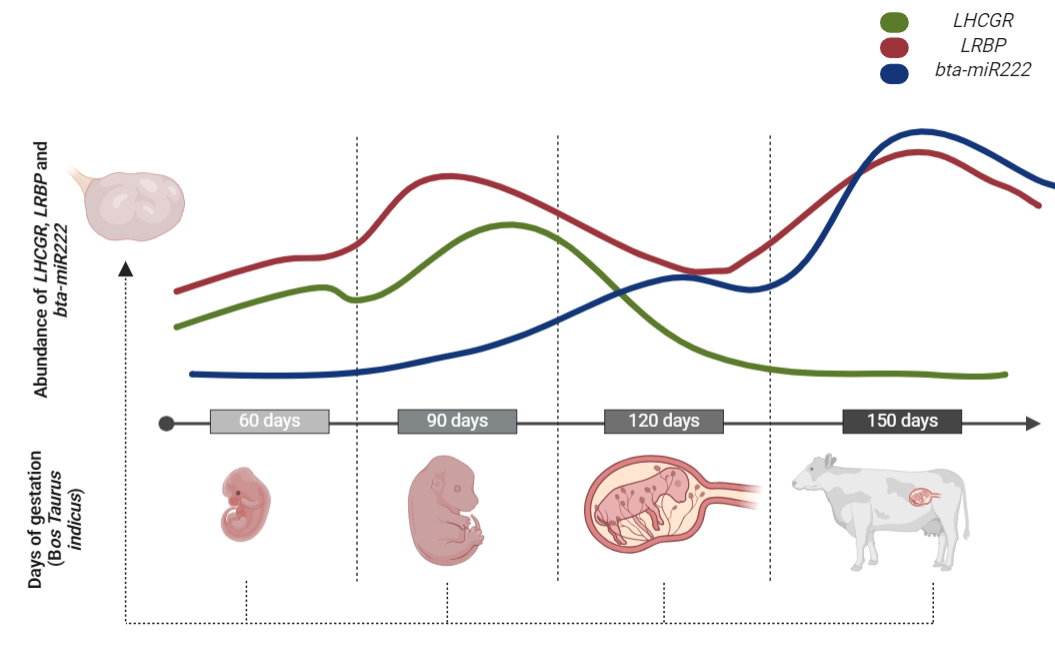
Experimental design along with the presentation of gene expression results for *LHCGR*, *LRBP* and *bta-miR222* in the bovine fetal ovary, organized according to gestational ages. Red line: *LRBP;* Green line: *LHCGR;* Blue line: *bta-miR222.*

The immunolocalization of LHR was demonstrated in the fetal ovary. The LHR protein was found in oogonia, primordial, primary, and secondary follicles (Figure 2A). Moreover, both oocyte and granulosa cells showed positive results for LHR immunostaining (Figure 2B). Another important finding was the stronger immunoreactivity of LHCGR in the ovarian cortex (Figure 2A) than in the medullar region, which also showed positive immunostaining for stromal cells and blood vessels (Figure 2B). We summarize the results in an experimental design (Figure 3).

## Discussion

For the first study, we describe the time-dependent expression patterns of *LHCGR*, *LRBP*, bta-miR-222 to reinforce the steroidogenic capacity of fetal ovary in cattle. LH plays a key role in the control of the physiological processes in the ovary, such as the development of antral follicles and ovulation, and acts via the LH receptor (Bao et al., 1997; Fortune, 2001; Nogueira et al., 2007; Xu et al., 1995). *LHCGR* mRNA has been detected in the preantral follicles of rats, rabbits, and pigs (Eppig, 2001), however, the location of LHCGR in bovine ovaries had previously, only been demonstrated in the adult and not in fetal bovine ovaries (Braw-Tal and Roth, 2005).

The transcriptional regulation of *LHCGR* can be influenced by several factors, particularly in granulosa cells, which are FSH-dependent (Nogueira et al., 2007). The data regarding the time at which the ovarian follicles acquire LHCGR in the granulosa cells are conflicting. Some authors have demonstrated that the dominant follicle acquires LHCGR before follicle deviation (Xu et al., 1995), but other reports show that *LHCGR* expression occurs after this step (Bao et al., 1997; Fortune, 2001; Xu et al., 1995). In the present study, we demonstrated that the LHCGR was present in oogonia, primordial, primary, and secondary follicles and that the expression was not exclusive from granulosa and theca cells, as described in bovine antral follicles (Fortune et al., 2001; Garverick et al., 2002; Nogueira et al., 2007; Xu et al., 1995). Therefore, we considered that *LHCGR* expression may be differentially regulated in the preantral follicle and modified when these follicles become gonadotropin dependent during the later stages of their differentiation.

Previous data showed the presence of gonadotropins receptors in the cumulus-oocyte complex of rats and cows (Amsterdam et al., 1976; Bodensteiner et al., 1996). In support of these results, (Baltar et al., 2000) described the positive and conclusive binding of LH or hCG to LHCGR in the bovine cumulus-oocyte complex. The same findings were described by previous studies, which observed that the cumulus cells of mice possessed LH receptors, although the quantities were smaller than those found in granulosa cells (Teerds and Dorrington, 1995). Our data on the expression of the LHCGR in fetal ovaries agree with (Teerds and Dorrington, 1995), and (Bao et al., 1997) who showed that the receptors were in cumulus cells, granulosa cells, and preantral follicles but disagree with Eppig (Eppig, 1991) who reported the absence of these receptors in these cells types, and (Braw-Tal and Roth, 2005) detected *LHCGR* only in inner theca cells.

The negative correlation between *LRBP* expression and *LHCGR* mRNA regulation, at the follicular deviation stage in cattle, was also reported by Ereno et al. (2015) The authors suggested that the lower abundance of *LRBP* mRNA in dominant follicles was consistent with the involvement of the LHCGR/LRBP system during follicle selection, to ensure the expression of *LHCGR* mRNA and the acquisition of ovulatory capacity. Similarly, we observed that the decreased abundance of *LHCGR* was accompanied by an increase in the abundance of *LRBP*, which suggested that LHCGR in the fetal ovary may be regulated by this protein; therefore, the higher abundance of *LRBP* on day 150 may contribute to the lower expression of *LHCGR* in the same period.

Post-transcriptional regulation by miRNA in the ovary has been reported in several species (Gilchrist et al., 2016; Menon et al., 2013). The miRNA expression and their specific roles in bovine ovary were previously reported (Zielak-Steciwko and Evans, 2016) including those of miR-222, which was described by (Hossain et al., 2009) as a possible regulator of *LHCGR* expression. In addition, (Salilew-Wondim et al., 2014) demonstrated that the expression of miR-222 in the theca and granulosa cells of bovine antral follicles was lower than that in granulosa cells from bovine dominant follicles. Recently, (Santos et al., 2018) demonstrated the expression of bta-miR-222 in adult and fetal bovine tissues (ovary, testicle, spleen, liver, kidney, heart, and brain); in addition showed that the abundance of *LHCGR* mRNA and the expression of bta-miR-222 followed opposite patterns in superstimulated granulosa cells from Nelore cattle. Here, the lower levels of *LHCGR* on day 150 of gestation could be supported by the upregulation of bta-miR-222; the higher expression may be required to regulate preantral follicle formation, especially during the secondary follicle formation at this time. Based on the findings discussed above, we propose the possibility of a summative effect between bta-miR-222 and *LRBP*, which promotes *LHCGR* downregulation in the developing bovine fetal ovary.

Although fetal ovarian development occurs independently of gonadotrophins, the findings regarding the differential expression of *LHCGR/LRBP* suggest that this system could be one more factor that must be regulated to allow the establishment of germ cells and fetal ovarian development. In addition, it could enable better strategies, using hormonal protocols, to take advantage of the reproductive potential of cattle.

## Conclusion

In summary, we suggest the involvement of LHCGR/LRBP regulation with mechanisms related to the development of preantral follicles. Furthermore, taken together, lower expression of *LHCGR* mRNA in bovine fetal ovaries on day 150 is negatively associated to higher expression of *LRBP* and bta-miR-222.
